# Assessment of Aqueous Extraction Methods on Extractable Organic Matter and Hydrophobic/Hydrophilic Fractions of Virgin Forest Soils

**DOI:** 10.3390/molecules26092480

**Published:** 2021-04-23

**Authors:** Wan Muhammad Ikram Wan Mohd Zamri, Fridelina Sjahrir, Nor Suhaila Yaacob, Noor Fazreen Dzulkafli, Mohd Fadzli Ahmad, Hasdianty Abdullah, Maegala Nallapan Maniyam, Emi Fazlina Hashim, Nobuyuki Kawasaki, Kazuhiro Komatsu, Victor S. Kuwahara

**Affiliations:** 1 Department of Science & Biotechnology, Faculty of Engineering & Life Sciences, Universiti Selangor, Bestari Jaya 45600, Selangor, Malaysia; ikramzamri1995@gmail.com (W.M.I.W.M.Z.); fridelina@unisel.edu.my (F.S.); fazreen@unisel.edu.my (N.F.D.); fadzli@unisel.edu.my (M.F.A.); dianty@unisel.edu.my (H.A.); hashim.emifazlina@nies.go.jp (E.F.H.); 2 Institute of Bio-IT Selangor, Universiti Selangor, Jalan Zirkon A7/A, Seksyen 7, Shah Alam 40000, Selangor, Malaysia; maegala@unisel.edu.my; 3 Centre for Foundation and General Studies, Universiti Selangor, Jalan Zirkon A7/A, Seksyen 7, Shah Alam 40000, Selangor, Malaysia; 4 Dainippon Ink and Chemicals DIC Corporation, Central Research Laboratories, 631 Sakado, Chiba 285-8668, Sakura, Japan; nobuyuki-kawasaki@ma.dic.co.jp; 5 National Institute for Environmental Studies, 16-2 Onogawa, Tsukuba 305-8506, Ibaraki, Japan; kkomatsu@nies.go.jp; 6 Faculty of Education & Graduate School of Engineering, Soka University, 1-236 Tangi-Machi, Hachioji-Shi 192-8577, Tokyo, Japan; victor@soka.ac.jp

**Keywords:** autoclaved, dissolved organic carbon, fractions, humic substances, soil extraction, total dissolved nitrogen, total dissolved phosphorus

## Abstract

The assessment of water-extractable organic matter using an autoclave can provide useful information on physical, chemical, and biological changes within the soil. The present study used virgin forest soils from Chini Forest Reserve, Langkawi Island, and Kenyir Forest Reserve (Malaysia), extracted using different extraction methods. The dissolved organic carbon (DOC), total dissolved nitrogen (TDN), total dissolved phosphorus (TDP), and ammonium-nitrate content were higher in the autoclave treatments, up to 3.0, 1.3, 1.2, and 1.4 times more than by natural extraction (extracted for 24 h at room temperature). Overall, the highest extractable DOC, TDN, TDP, ammonium and nitrate could be seen under autoclaved conditions 121 °C 2×, up to 146.74 mg C/L, 8.97 mg N/L, 0.23 mg P/L, 5.43 mg N mg/L and 3.47 N mg/L, respectively. The soil extracts became slightly acidic with a higher temperature and longer duration. Similar trends were observed in the humic and nonhumic substances, where different types of soil extract treatments influenced the concentrations of the fractions. Different soil extraction methods can provide further details, thus widening the application of soil extracts, especially in microbes.

## 1. Introduction

Dissolved organic matter (DOM) is ubiquitous in aquatic environments and plays a critical role in the interplay between the hydrosphere, biosphere, and atmosphere [1]. Water is the standard and most widely used solvent, and water-extractable organic matter is considered a readily available substrate in soil extraction for the preparation of enriched soil-media for algae cultivation [2,3]. Even though the extraction comprised only <2% of the total soil organic matter, the process signified important roles in many chemical and biological processes in topsoil and subsoil [4]. Soil organic matter (SOM) is the largest carbon pool in the terrestrial ecosystem [5]. SOM can exist in different forms of fractions as simple as amino acids and monomeric acids to complex molecules such as protein, lignin, cellulose, and it all comes together with decomposed and partially decomposed plant and microbial residues [6]. Numerous studies have focused on carbon cycle modelling to improve the understanding of carbon stabilization in soils [5,7,8].

The organic component of soil can be divided into SOM and DOM. DOM is identified to include a broad spectrum of organic constituents with molecular weights ranging from hundreds to more than 300,000 Da [9]. It represents a small but highly important fraction of the SOM carbon due to its high reactivity [10]. DOM is a mixture of various organic aromatic compounds derived from fulvic acids, lignin, oligomeric and monomeric sugar derivatives, amino acids, and fatty acids between C14 and C54 originating from plant and dead bacteria [4,11]. Two major types of DOM can be identified as nonhumic and humic substances. Nonhumic substances are the compounds of well-described classes such as amino acids, lipids, proteins, waxes, and carbohydrates [12]. Humic substances are supramolecular (a complex of molecules), rich in oxygen-containing functional groups, commonly COOH, phenolic and enolic OH, alcoholic OH, and C=O of quinones [13]. However, DOM and DOC are used differently when the dissolved fraction of organic matter is used. DOM can be defined as the entire organic molecule, while DOC is only specific to the carbon fraction. Since DOM is difficult to quantify, carbon measurements are preferred [14]. However, DOM can be assumed as 45–50% organic carbon by mass [15]. The presence of humic substances greatly influences the natural organic material, as they are very soluble and provide complex groups such as carboxylic and phenolic hydroxyls [16]. They form stable complex compounds and act as carriers for toxic metals, vital for the bioavailability and mobility of metals in soil, sediment, and aquatic systems.

Besides water, commonly used solvents are NaOH, Na_4_P_2_O_7_, EDA (ethylenediamine), DMSO (dimethylsulfoxide), and other organic solvents that can dissolve insoluble organic matter under natural condition [17]. However, these solvents contribute to the alteration of the chemical characteristics of the extracted DOM. NaOH and Na_4_P_2_O_7_ stimulate the oxidation process, while EDA enriches the recovery nutrients with nitrogen [18]. Thus, water, a natural solvent, should be used to limit the chemical alteration of extracted SOM [19]. However, water is less efficient than other solvents in extracting a high amount of DOM because the complex humus hinders the ability of water to solubilize soil nutrients. Previous research has demonstrated a wide range of extraction temperatures (20 °C to 100 °C), with extraction times from 1 h to 24 h [19]. However, the possibility of aqueous soil extraction methods using an autoclave has been overlooked. Hence, the purpose of our study was to evaluate the effect of elevated temperature and extraction times on the chemical composition of organic matter in water extraction. This study focuses on the concentrations of hydrophobic and hydrophilic fractions of soil organic matter using the designated extraction methods.

## 2. Results

### 2.1. Physicochemical Analysis of Soils

The physicochemical analysis of soils is recorded in Table 1 and Table 2. All soils are distinguished as acidic (low pH ranging from 3.97 to 4.83). The texture of Chini lake and Kenyir soils are composed of silt (94.89–94.91%), sand (4.47–4.57%), and clay (0.52–0.63%). Meanwhile, the Langkawi Island soil is mainly composed of sand (95.00%), silt (0.00%), and clay (4.00%). The obvious physical observation can be made based on the soil color, where Chini was dark brown, while Kenyir and Langkawi Island soils were light yellowish-brown. The color of soils can be an indicator of the availability of organic matter content in the soil.

The chemical contents in the soil are listed in Table 2. Chini soil exhibited the highest concentrations of organic matter in most parameters, i.e., soil organic carbon (SOC), nitrogen (N), and phosphorus (P) as 1.03–55.78%, 0.11–0.41%, and 1.62–24.23%. Meanwhile, no significant differences (*p* < 0.05) are noted in the Langkawi and Kenyir soils in terms of total carbon (C), N, and P content. The heavy metal results vary, and some parameters could not be determined due to the very low (out of range) concentrations. The level of iron (Fe) ranged from 755.47 to 771.98 mg/kg, with no significant difference (*p* < 0.05) between the soils. Kenyir soil recorded very high concentrations of lead (Pb), cadmium (Cd), and zinc (Zn) at 33.83, 0.27, and 27.09 mg/kg but the levels of arsenic (As), mercury (Hg), and silver (Ag) cannot be determined due to concentrations fall below range of measurement.

### 2.2. Chemical Characterization of Different Aqueous Soil Extraction Methods

Table 3 summarizes the results of selected chemical parameters using five different extraction methods. In general, autoclaved soil extracts always showed higher extractable nutrients than for 24 h room temperature extraction (*p* < 0.05). The percentage of DOC extracted using autoclaved temperature, 105 °C 1×, ranged from 195.67% to 331.85%. In comparison, the autoclaved extract at 121 °C 1× exhibited a significantly higher percentage (414.94–669.44%) than at 105 °C 1×. Total dissolved nitrogen (TDN) also showed a similar trend, where the autoclaved temperature at 121 °C 1× always yielded a higher percentage of nitrogen (89.52–161.30%), which was significantly different to the autoclaved temperature at 105 °C 1× (31.88–114.57%). Extractable total dissolved phosphorus (TDP) was also significantly higher when autoclaved at 121 °C 1× (46.88–1800%) than at 105 °C 1× (40.63–1175%). Similarly, the ammonia content showed the highest percentage, ranging from 98.10–204.15% when autoclaved at 121 °C 1× than at 105 °C 1×, ranging from 35.24–84.33%, which was significantly lower (*p* < 0.05). 

Another approach to aqueous extractable organic matter of soil focuses on autoclave duration at different temperatures, 105 °C and 121 °C. Prolonging the autoclave process duration from 1 h to 2 h increased the amount of extractable nutrient slightly, whereby an enhanced extractable percentage of DOC (21.70–37.76%) was noted (Table 3). The results indicate that prolonged autoclave duration from 1 h to 2 h did not significantly increase or decrease the TDN, except for autoclaved Chini lake soil at 121 °C. The extractable amount of TDP significantly increased upon the prolonged duration of the autoclave process at 121 °C (41.73–44.68%), except for Langkawi soil, where both autoclave temperatures did not enhance extractable TDP. Next, autoclaving at 121 °C showed a significant increase in three soil samples ranging from 20.7–81.73%. Autoclaving at 105 °C did not show any significant enhancement except for the Chini soil, which showed a percentage increase of about 34.50%. Meanwhile, the Chini and Langkawi soils showed a significant increase at 105 °C and 121 °C (37.58–41.17%) for nitrate, but not in the Kenyir soil. 

**Figure 1:**
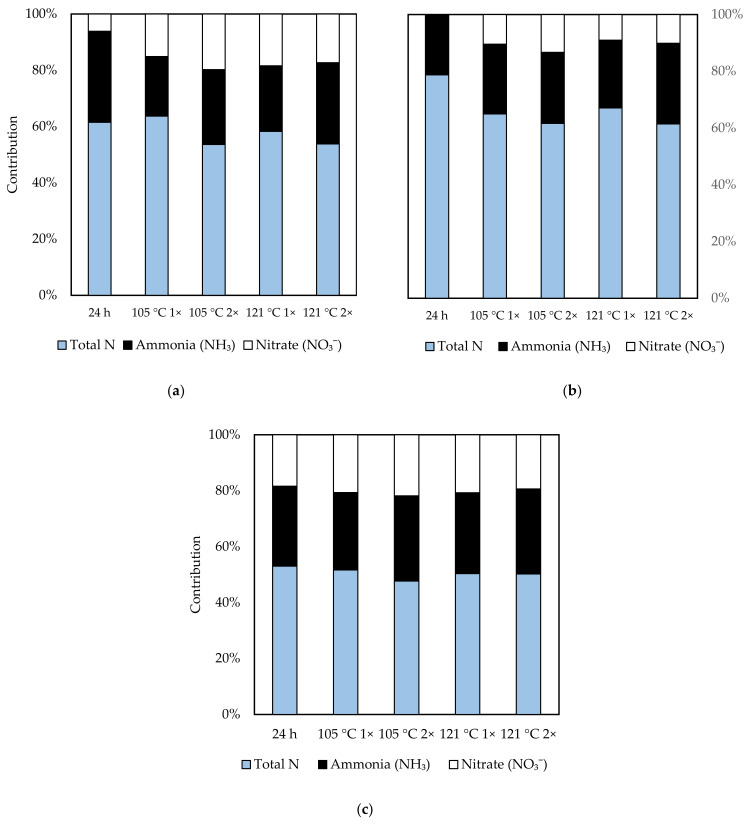
Total contribution of ammonia, NH_4_^+^ and nitrate, NO_3_ to total organic N for (**a**) Chini Forest Reserve (**b**) Langkawi Island and (**c**) Kenyir soil, respectively. Bars represent average value of concentrations at (*n* = 3).

Concentrations of total N in soil extracts were dominated by ammonia (NH_3_) in all soil samples (Figure 1). NH_3_ contributed to 27% to 54% and 33% to 74% of total N for natural extractions and autoclaved extractions, respectively. Nitrate concentrations contributed to about 10% to 34% of total N for natural extractions. Meanwhile, the nitrate amount for autoclaved extraction increased to some extent, i.e., from 16% to 54% of total N. 

**Figure 2:**
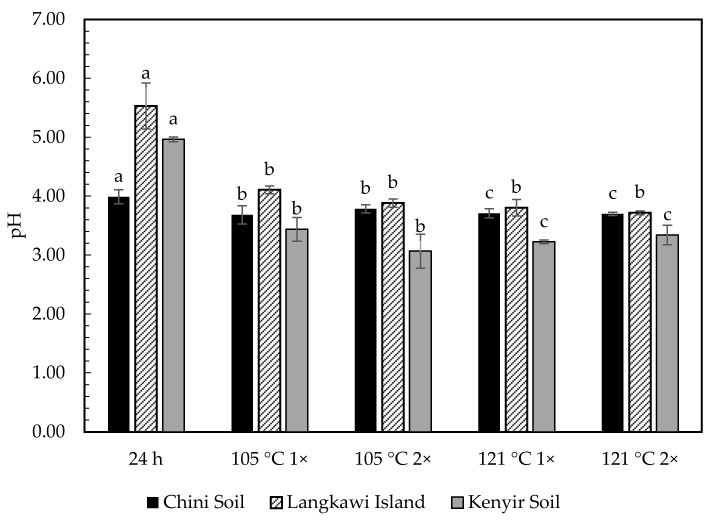
The pH of different extraction methods. Bars on the graph represent the standard error at *n* = 3. ^a,b,c^ Same letter on top of graph indicated significant difference using one-way ANOVA (*p* > 0.05) and Tukey post hoc. Comparison between treatments; (24 h—105 °C 1×, 24 h—121 °C 1×, 105 °C 1×–105 °C 2× and 121 °C 1×–121 °C 2×).

Figure 2 shows the significant difference of in the pH of the soil using natural extraction compared to the autoclave extraction, except for Langkawi soil, where the pH changes were unnoticeable. When autoclaved, the soil extract became more acidic (lower pH value). Although increasing the autoclave duration from 1 h to 2 h decreased the pH value slightly, the value was too small to notice.

In general, the autoclaved extracts always showed a higher amount of extractable DOM than the 24 h incubation at room temperature. Moreover, autoclaving at 121 °C exhibited the highest range of DOM achieved. The prolonged autoclave duration of aqueous soil extracts at 121 °C 2× achieved the highest amount of extractable DOM than other treatments, although the trend indicated various patterns among the soils tested.

### 2.3. Hydrophobic and Hydrophilic Fraction

**Figure 3:**
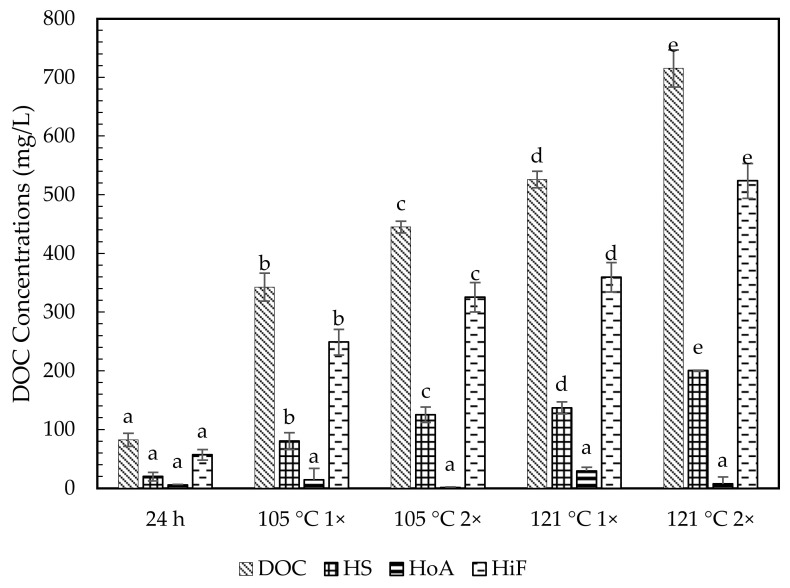
Fraction of dissolved organic matter for Chini soil extracts. DOM fractions are DOC, total dissolved organic carbon; HS, humic substances; HoA; hydrophobic neutral and HiF, hydrophilic fraction. Bars represent standard error with *n* = 3. Same letter on top of graph indicated no significance difference using one-way ANOVA (*p* > 0.05) and Tukey post hoc. Comparison between treatments; (24 h—105 °C 1×, 24 h—121 °C 1×, 105 °C 1×–105 °C 2× and 121 °C 1×–121 °C 2×).

**Figure 4:**
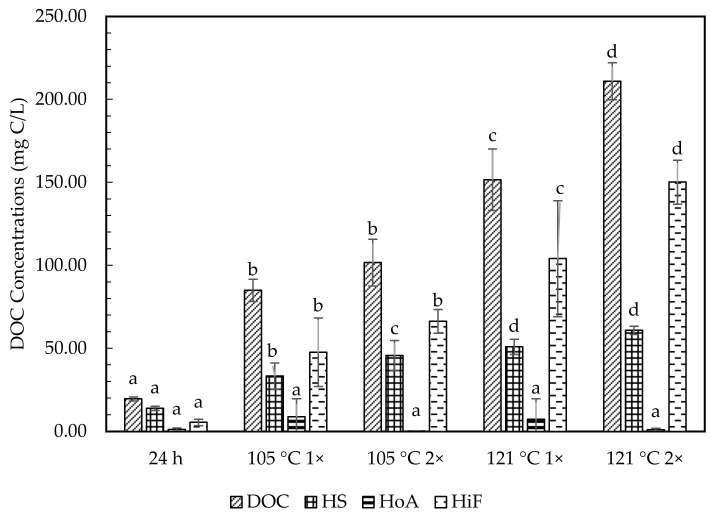
Fraction of dissolved organic matter for Langkawi soil extracts. DOM fractions are DOC, total dissolved organic carbon; HS, humic substances; HoA; hydrophobic neutral and HiF, hydrophilic fraction. Bar represents standard error at *n* = 3. Same letter on top of graph indicated no significance difference using one-way ANOVA (*p* > 0.05) and Tukey post hoc. Comparison between treatments; (24 h—105 °C 1×, 24 h—121 °C 1×, 105 °C 1×–105 °C 2× and 121 °C 1×–121 °C 2×).

**Figure 5:**
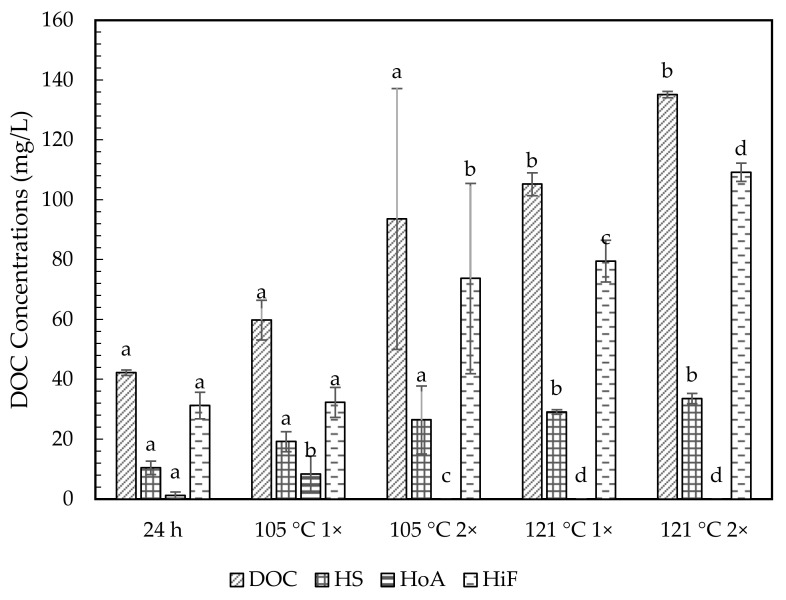
Fraction of dissolved organic matter for Kenyir soil extracts. DOM fractions are DOC, total dissolved organic carbon; HS, humic substances; HoA; hydrophobic neutral and HiF, hydrophilic fraction. Bars represent standard error with *n* = 3. Same letters on top of graph indicate no significance difference using one-way ANOVA (*p* > 0.05) and Tukey post hoc. Comparison between treatments; (24 h—105 °C 1×, 24 h—121 °C 1×, 105 °C 1×–105 °C 2× and 121 °C 1×–121 °C 2×).

Table 4 and Figure 3, Figure 4 and Figure 5 show the chemical characterization of the soil extract fractions. In general, the hydrophilic fraction (HiF) dominated in all soil extract treatments, with the percentage accounting for more than 54% of their DOM as DOC. Humic substances (HS) were the second most abundant fraction, ranging from about 23–32%. All treatments of soil extracts were mainly in acidic conditions. Meanwhile, the hydrophobic neutral fraction (HoN) was considered very low (0–6%) of the total DOM. The HoN value greatly declined in number (74% to 100%) upon prolonged autoclave duration. Overall, Chini soil contained the highest HS and HiF fractions, averaging between 19.86–200.25 mg C/L and 57.08–506.69 mg C/L. In contrast, the HS and HiF soil fractions for Langkawi ranged between 45.63–60.79 mg C/L and 66.27–150.13 mg C/L. The Kenyir DOM fraction indicated the lowest range from 10.43 to 33.57 mg C/L for HS and 31.2 to 109.20 mg C/L for HiF.

The extractable fraction DOM at room temperature showed a lower range of HS and HiF fractions, ranging from 10.43–19.86 mg C/L and 31.25–57.08 mg C/L. Meanwhile, both autoclave temperatures (105 °C and 121 °C) showed increasing DOM fractions in DOC, i.e., about 83–588% and 3–530% for HS and HiF. The autoclave temperature at 121 °C indicated a higher DOM fraction content, i.e., 1.4–2.4 times higher than 105 °C. The prolonged autoclave duration from 1 h to 2 h exhibited a greater increment for both HS and HiF fractions. The autoclave temperature of 105 °C denotes the increased percentage of about 37–55% for HS fraction and 30–128% for HiF. Apart from that, 121 °C 2× always showed the highest DOM fractions, with increased percentage (15–46% for H and 37–45% for HiF), except for HoN.

## 3. Discussion

### 3.1. Physicochemical Analysis of Soils

Based on the study by [20], the average pH of the three types of soil (inland, seasonal flood, and riverine) of the Chini forest ranged from 3.60 to 4.24, 3.80 to 4.37, and 3.82 to 4.16. The pH indicated that most of the soils from their study were acidic, and it is very common due to weathering and the leaching process occurring within the soil. Other than that, the decomposition of organic matter due to biological process also contributed to the soil acidity [21].

The solubility, bioavailability, and mobility of trace elements are controlled by soil pH; thus, affecting translocation in plants. Soil pH is also largely influenced by the partition of elements between the solid and liquid phases through precipitation−dissolution reactions [22]. It causes the mineral and organic soil fraction depending on the pH charges, where negative charges dominate in high pH and positive charges exist in low pH. At low pH, trace elements become soluble due to high desorption and low adsorption. This phenomenon could be explained by the competition between cation and the exuberance of H^+^ ions for available permanent charged sites restricting the sorption of potentially toxic metal cations onto these sites at low pH values. In contrast, at high pH values, the competition becomes feeble; thus, more metal is adsorbed [23,24]. In other words, the solubility of most trace metals becomes limited, leading to their low concentrations as the soil pH becomes higher [25]. In [26], they demonstrated the solubility of Zn at different levels of pH. At pH 7, only 1 mg/L of Zn of the 1200 mg/kg total Zn was present in soil solution, while at pH 6 and 5, the concentrations of Zn in soil solution were observed at 100 mg/L and 40 mg/L. From the current study, the Chini soil (pH 3.97) and Langkawi soil (pH 4.23) exhibited low concentrations of Pb and Zn, while the Kenyir soil (pH 4.83) contained higher concentrations of Pb and Zn. Besides adsorption, soil pH is also affected by the precipitation of carbonates, chlorides, phosphates, hydroxide, and sulphates. The application of lime and apatite causes high pH, directly decreasing Cu and Cd concentrations [27]. 

Soil quality indicators are physical, chemical, and biological properties that can be assessed to monitor soil changes [28,29]. The color of soils indicates the availability of soil organic matter, i.e., organic-mineral complexes. Commonly, dark soil contains more humus than light soil. Andosols (dark soils) are characterized by a low L* value, indicating higher C, N, and Fe concentrations. In contrast, yellowish soil has a higher L* value, representing lower C, N, and Fe [30].

### 3.2. Extractable Organic Matter Using Autoclaved Methods

Extraction technologies are often based on temperature and pressure strength using organic or aqueous solvents [31]. Bioactive compounds exhibit varying polarity, solubility, and chemical stability; thus, the appropriate pressure and solvent type should be selected to extract targeted bioactive compounds [32]. Temperature is another parameter that influences the extraction process. More organic matter could be extracted when autoclaved than from 24 h room temperature extraction. This is due to the disintegration of polysaccharide structures, alteration processes that influence the soil surface, and unknown reactions or nutrients within the soil matrix. Thus, the dissolved organic matter and ion concentrations could be enhanced in the liquid form [33]. Incubation at 20 °C from 1 h to 24 h showed minimal changes in extractable organic carbon [34]. The author also found that increasing the temperature could narrow the C:N ratio, indicating a higher composition of extractable organic nitrogen and NH_4_. A shorter extraction duration at room temperature was more desirable as the amount of extractable C and N did not change drastically. The carbohydrate and N (mineral) concentrations were significantly higher than with the longer extraction time [34]. Soil weathering is usually a slow process at low temperature (~28 °C) and might not significantly change within a day [35]. 

The low temperature might not be sufficient to extract high OM because the heat energy is too low to surpass the activation energy and allows the disrupted molecules to be released to the extracts [36]. Water-extractable organic carbon and nitrogen indicated a huge difference between 45 °C and 90 °C, exhibiting a constant exponential profile in response to the increasing temperature [37]. The extracted DOM yield could be enriched by the elevated temperature [33,38]. Besides, extractable organic carbon can be enhanced by the autoclaving processes [39,40]. The incidence of highly extractable organic matter at the autoclave temperature of 121 °C is probably due to the disintegration of heat to the stable compounds (C, N, P, and other compounds), releasing them into the extract. This process surpasses the activation energy of certain molecules to cleave the chemical bonds [41,42]. 

Total organic N was contributed the most by NH_4_^+^ (about 27–74%), followed by NO_3_ (10–54%). Based on a study by [43], the concentration of free N in soil extracts was dominated by NH_4_^+^, with 54–72% of total N, while NO_3_ contributed about 1–4% total N in forest and grassland soils. In [44], it was suggested that the increase of extraction temperature might promote the breakdown of organic N to more soluble molecules such as peptides, amino acids, and amino sugars, creating a pool rich in these molecules.

The pH changes in the autoclaved soil extract were probably due to the release of organic acids from the DOM pool, and the data was supported by the spectrum using C-CP/MS spectra, where the carboxylic peak declined [33]. The immensity of the decrease can be seen upon the soils’ initial acid-base conditions [45]. Two different studies reported that autoclave could reduce the pH of clay soil, whereas the other study observed no pH changes in silt loam soils [46]. Since the initial soils were already in acidic form, the results were expected. In contrast, Eutric Fluvisol soil with an initial pH of 7.44 changed to 7.77 after double steam sterilization due to the release of bases from the organic matter [47].

### 3.3. Hydrophobic and Hydrophilic Fractions of Soil Extracts 

Our results suggested that the concentrations of humic and nonhumic substances could be increased by autoclaving and prolonging the process duration, supported by the analysis obtained from Table 3 and Table 4. The carbon content in humic acid from four different Mumbai regions ranged from 17% to 53% [48]. Meanwhile, [49] conducted a thermodynamics stability study of humus at 10, 20, 40, and 55 °C. The author observed that the transformation of fulvic acids to humic acids were significantly higher at 40 and 55 °C. The authors of [50] reported considerable changes were observed in the UV−Vis spectra (280 nm) of humic acid solutions at a temperature of 95 °C for 24 h storage than at 80 °C. The study also compared the storage time at 24 h and a week, where the substantial changes observed at 95 °C for 24 h was comparable to the storage time for 1 week at 80 °C. The temperature-dependent changes signified more condensed polyaromatic structures by condensing phenolic and carboxylic groups of the humic acids [51]. Humic acids under higher temperature and extended durations underwent irreversible changes at 80 °C through water loss. At 95 °C and above, the changes were probably due to the release of chemisorbed water molecules or water molecules from the condensation process, which could exist among the adjacent carboxylic and alcoholic groups of humic acids [50]. In [52] and [53] it was revealed that the humic fraction became the main organic component with 2- to 3-fold increase upon increased heating times at <250 °C. The study by [53] also concluded that at high temperature (300 °C), humic fraction decreased with increased heating time, and at 200 °C, the proportion of the humic fraction increased with increased heating time. The main factor in humic structure changes was demonstrated by [54,55], i.e., structural changes at a high temperature (350 °C) were due to various factors, including increased aromatic content, removal of oxygen-containing functional groups, heterocyclic formation N compounds, and the reduction of the chain length of alkyl compounds. The ^13^C NMR spectra results showed that unheated soils contained high alkyl and O-alkyl with low aromatic and carboxyl C contents, while heated samples contained high aromatics and carboxyl C with low alkyl and O-alkyl. The basic structure of humic acids remained unchanged up to 250 °C, and decarboxylation occurred above 300 °C. These studies concluded that the increase of temperature and heating duration might extract more humic substances without altering their structure.

## 4. Materials and Methods

**Figure 6:**
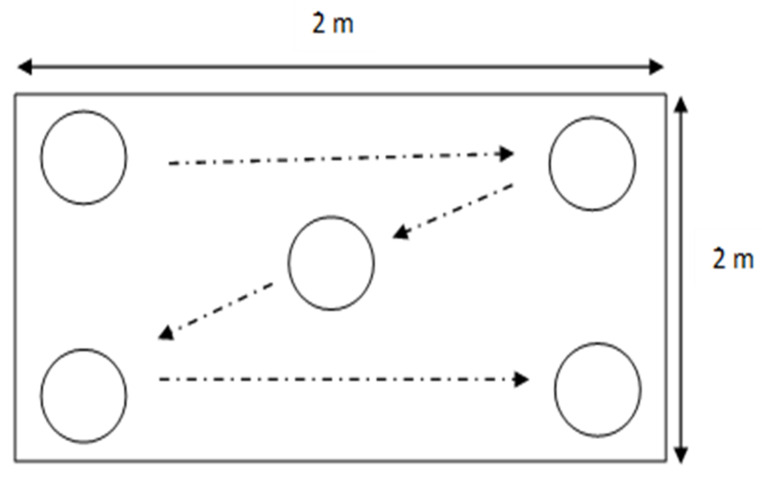
Random sampling method at 5 different points in a designated area [53].

Three mineral soil samples from Peninsular Malaysia were collected for this study, Sg. Beruang near Chini Lake, Pahang (3°26′01.3″ N 102°55′08.0″ E), Singa Besar Island, Langkawi Island, Kedah (6°12′51.2″ N 99°44′47.9″ E) and Sah Kecil Island, a small island located at Kenyir Lake (5°05′01.7″ N 102°48′08.6″ E). The location of sampling sites was illustrated based on Figure S1. The random technique was conducted for soil sampling, according to the United State Department of Agriculture (USDA) method suggested by [56]. Figure 6 illustrates the process of soil collection. Soil surface or O horizon of about 15 cm from the soil surface was removed before the sample was collected. Five cores with uniform characteristics were collected about 1 kg each before being homogenized into one composite. Coarse particles such as stones, wood, and roots were removed, and the samples were dried at 60 °C until moisture was completely removed. Upon completion, the dried soils were ground to powder and sieved using a 2-mm stainless steel sieve. The selected soil characteristics and analytical method are shown in Table 5.

### 4.1. Soil Extraction

The extraction process was carried out in triplicate. Aqueous extraction was done according to previous studies with slight modification [57,58]. Five different aqueous extraction treatments were conducted (Table 6). Approximately 20 g of each processed soil sample was put into Schott bottles with 200 mL ultra-pure water (1:10) and hand-shaken vigorously (end to end) for 5 min. For the room temperature extraction, samples were incubated for 24 h (24 h) in the dark. Meanwhile, for the high-temperature aqueous extraction, the autoclave SX-500 (Tomy Seiko Co., Ltd., Tokyo, Japan) was used. The samples were autoclaved for 1 h and 2 h with a slight change of temperature, i.e., 105 °C and 121 °C. Details about the procedure are listed in Table 6. After incubation, the bottles were hand-shaken for 1 min to resuspend the soil. The samples were centrifuged using the Beckman Allegra X-30R (Beckman Coulter, Indianapolis, IN, USA) centrifuge at 2500 rpm for 15 min. The supernatant was filtered using 0.7 µm Whatman Glass Microfiber Filter (GF/F) (Cytiva, Maidstone, United Kingdoms), and the filtrate was stored at −20 °C in Revco, ULT-390-10 freezer (Thermo Fisher Scientific, Waltham, Massachusetts, United States) for further analysis.

### 4.2. Macronutrient Analysis

Total dissolved nitrogen (TDN) and total dissolved phosphorus (TDP) of the extracts were measured using a portable spectrophotometer, Lovibond MD600 (The Tintometer Limited, Amesbury, UK). Each parameter was measured using designated reagents and procedure according to the given instructions in the manual. Dissolved organic carbon (DOC) of extracts was measured as nonpurgeable organic carbon (NPOC) using Shimadzu TOC-L CSH (Shimadzu, Kyoto, Japan) The sample was transferred into a 9 mL vial, and 1% of the total volume of the sample was added with 2 M hydrochloric acid (HCl) to remove volatile carbon. Since the NPOC was measured using high sensitivity catalyst, manual dilution of the concentrated samples was done. Triplicate samples (*n* = 3) for each treatment were measured for statistical analysis. 

### 4.3. Hydrophobic and Hydrophilic Fraction

**Figure 7:**
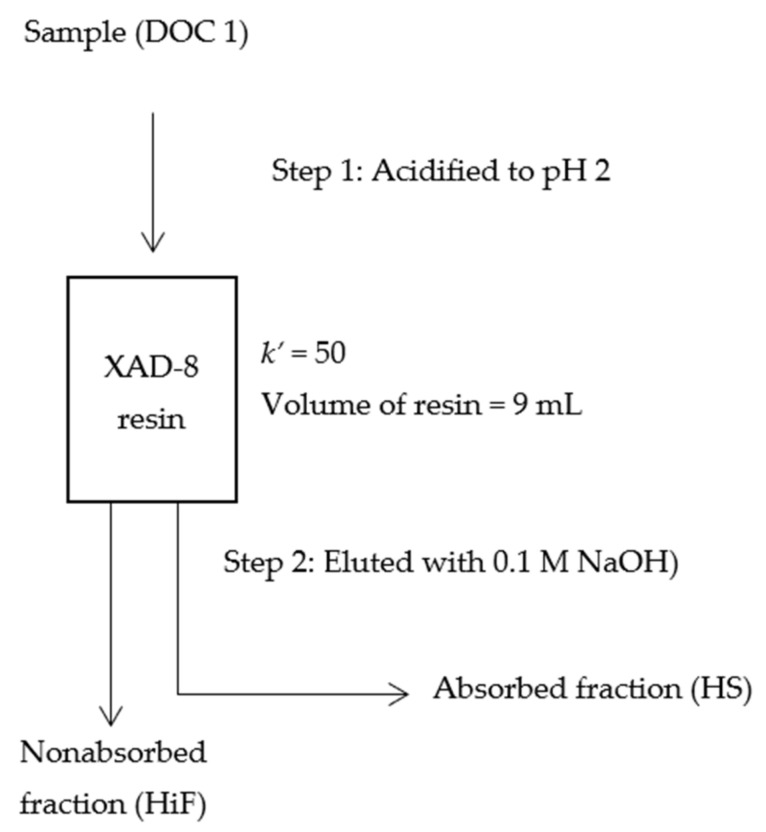
Experimental procedure for separation into absorbed fractions and unabsorbed fractions using soil samples. Details procedures explained and described in Figure 8.

**Figure 8:**
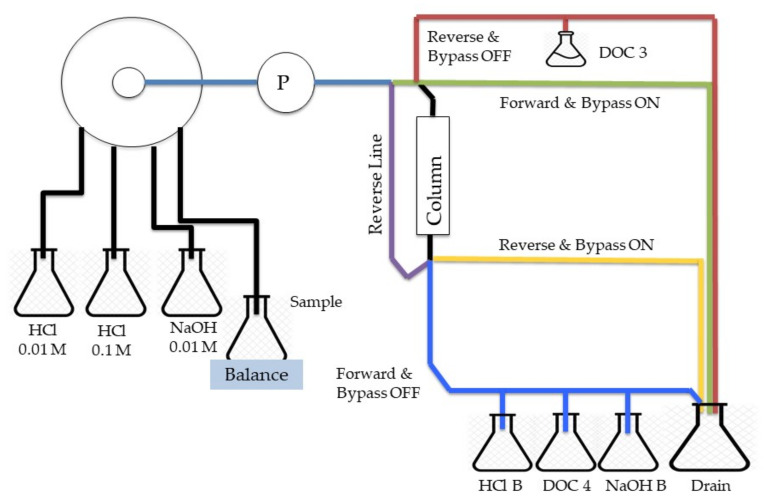
Schematic of Auto Fractionation System.

In this study, the DOM fractionation was conducted based on hydrophobic and hydrophilic interactions using ion-exchange resins (XAD-8 resins, obtained from National of Environmental Studies, Tsukuba, Ibaraki, Japan). DOM fractionation was obtained by a two-step procedure described in Figure 7 [59,60]. Fractionation of samples was described based on hydrophobic (absorbed, known as HS) and hydrophilic (nonabsorbed, known as HiF) fractions. All glassware used in this operation was precombusted at 450 °C for 4 h. In the first step, DOM 1 (sample) was acidified to pH 2 using 6 M HCl and then pumped through the Amberlite XAD-8 resin (20–60 mesh) using a peristaltic pump with Tygon tubing. The column was rinsed with a 1–2 bed volume of 0.1 M HCl to elute the HiF. HS was then eluted with 0.1 M of sodium hydroxide (NaOH). Both HiF and HS were collected and stored in Schott bottles. Figure 8 shows the schematic system of the auto fractionation system used in this experiment.

In this experiment, the column capacity factor *k′* through XAD-8 resin was set at 50. Factor *k′* can be defined as the ratio between the sum of organic matter absorbed onto XAD resin/the sum of organic matter not absorbed onto XAD resin. Every organic matter has its own *k′* factor and breaking the hydrophobic/hydrophilic fraction could be defined in a situation where 50% of organic matter with *k′* = 50–100 was absorbed onto XAD resin. Two different experiments were conducted where [61] used *k′* = 50, and [62] used *k′* =100. So, *k′* has been used as the parameter for defining HS in the fractionation process. The following equation has been suggested by [59] regarding the relationship between sample volume and *k′*:

(1) $$k\prime =\frac{V_{\mathrm{el}}}{2V_{0}}-1$$

where,

V_el_ = sample volume
V_0_ = volume of XAD-8 resin (65% bulk column volume)

In this experiment, the volume of XAD-8 resin was measured at 9 mL and *k′* = 50. Therefore, the calculation of total volume of sample should be:

V_el_ = (50 + 1) × (2 × (9 mL × 0.65)) (2)

### 4.4. Statistical Analysis

All results were analyzed using one-way analysis variance (ANOVA) and Tukey post hoc at *p* < 0.05. The significant difference between treatment was calculated using SPSS Software at a 95% interval level. All samples were analyzed in triplicate to obtain the average values for each analysis.

## 5. Conclusions

In conclusion, the results suggest that the composition of extractable organic matter varies using different extraction methods, and the recovery of organic matter depends on the initial conditions of soil and heat provided during the autoclaving process. Soil extracts using the autoclave yielded more DOC, TDN, TDP and ammonium nitrate compared to natural extraction, up to 3.0, 1.3,1.2 and 1.4 times higher, respectively. Nevertheless, the autoclave treatment can be proposed as a standard procedure to extract high organic matter, at least in this study. The best treatment in terms of the highest extractable organic matter was noted in the 121 °C 2× autoclave approach. A higher fraction of soil extracts can also be recovered in autoclaved soil without altering the basic structure of humic and nonhumic substances. The recovered organic matter information can be utilized to understand its various applications, especially as a growth promoter for targeted microorganisms. The study could also reduce the cost and increase the effectiveness of existing artificial media for microbial growth.

## Figures and Tables

**Table 1:** Physical properties of soils.

Soil Sample	Chini Lake	Langkawi Island	Kenyir Lake
pH	3.97 ± 0.03 ^a^	4.23 ± 0.03 ^b^	4.83 ± 0.03 ^c^
Sand (%)	4.47 ± 0.06 ^a^	95 ± 0.00 ^b^	4.57 ± 0.00 ^a^
Silt (%)	94.89 ± 0.06 ^a^	0.00 ± 0.00 ^b^	94.91 ± 0.00 ^a^
Clay (%)	0.63 ± 0.01 ^a^	4.00 ± 0.00 ^b^	0.52 ± 0.00 ^c^
Soil type	Silt loam	Sandy loam	Silt loam
Color	Dark brown	Light yellowish brown	Light yellowish brown

The value shows as mean value with (*n* = 3) followed by standard deviation, ±. ^a,b,c^ within rows, mean values followed by the same letter are not significantly different according to one-way ANOVA (*p* > 0.05) and Tukey post hoc.

**Table 2:** Chemical Analysis of soils.

Soil Location	Chini Lake	Langkawi Island	Kenyir Lake
Total Organic Carbon (%)	55.78 ± 1.29 ^a^	2.34 ± 0.02 ^b^	1.03 ± 0.02 ^b^
Nitrogen (%)	0.41 ± 0.05 ^a^	0.17 ± 0.00 ^b^	0.17 ± 0.00 ^b^
Phosphorus (%)	24.23 ± 0.84 ^a^	2.27 ± 0.01 ^b^	1.62 ± 0.01 ^b^
**Trace Metals (mg/kg)**			
Iron	755.47 ± 16.15 ^a^	767.09 ± 1.57 ^a^	771.98 ± 2.21 ^a^
Arsenic	ND < 1	ND < 1	ND < 1
Cadmium	0.20 ± 0.03 ^a^	0.25 ± 0.03 ^a^	0.27 ± 0.08 ^a^
Chromium	34.45 ± 3.92	ND < 2	ND < 2
Chromium VII	2.98 ± 0.18	ND < 3	ND < 3
Lead	3.06 ± 0.31 ^a^	3.52 ± 0.23 ^a^	33.83 ± 1.90 ^b^
Copper	5.26 ± 0.43 ^a^	1.23 ± 0.08 ^b^	1.65 ± 0.03 ^b^
Nickel	5.50 ± 0.33 ^a^	8.98 ± 0.54 ^b^	5.42a ± 0.29 ^a^
Zinc	5.67 ± 1.05 ^a^	8.56 ± 0.45 ^b^	27.09 ± 1.07 ^c^
Mercury	ND < 1	ND < 1	ND < 1
Silver	ND < 10	ND < 10	ND < 10

ND = not determined; ^a,b,c^ within rows, mean values followed by same letter are not significantly different according to one-way ANOVA (*p* > 0.05) and Tukey post hoc.

**Table 3:** Selected chemical analysis of soil extracts using various extraction methods.

Site	Treatments	Dissolved Organic Carbon, C (mg/L)	Total Dissolved Nitrogen, N (mg/L)	Total Dissolved Phosphorus, PO_4_ (mg/L)	Ammonia Concentrations, NH_3_ (mg/L)	Nitrate, NO_3_− (mg/L)
Chini Forest Reserve	24 h	74.15 ± 5.21 ^a^	6.63 ± 0.3 ^a^	0.20 ± 0.03 ^a^	3.50 ± 0.53 ^a^	0.67 ± 0.58 ^a^
	105 °C 1×	303.22 ± 17.19 ^b^	14.23 ± 1.65 ^b^	0.67 ± 0.3 ^b^	4.73 ± 0.12 ^b^	3.40 ± 0.61 ^b^
	105 °C 2×	404.48 ± 24.11 ^c^	12.80 ± 1.9 ^b^	0.73 ± 0.2 ^b^	6.37 ± 0.21 ^c^	4.73 ± 0.40 ^c^
	121 °C 1×	503.11 ± 39.99 ^d^	17.33 ± 2.0 ^b^	1.04 ± 0.06 ^c^	6.93 ± 1.01 ^d^	5.50 ± 0.78 ^d^
	121 °C 2×	677.95 ± 69.10 ^e^	23.47 ± 4.0 ^c^	1.47 ± 0.17 ^d^	12.60 ± 0.40 ^e^	7.57 ± 0.32 ^e^
Langkawi Island	24 h	19.92 ± 1.26 ^a^	5.33 ± 1.89 ^a^	0.01 ± 0.02 ^a^	1.45 ± 0.07 ^a^	NA
	105 °C 1×	86.03 ± 4.62 ^b^	7.03 ± 0.15 ^a^	0.17 ± 0.01 ^b^	2.67 ± 0.23 ^b^	1.13 ± 0.15 ^a^
	105 °C 2×	108.55 ± 5.61 ^c^	7.37 ± 0.23 ^a^	0.21 ± 0.05 ^b^	3.00 ± 0.20 ^b^	1.60 ± 0.10 ^b^
	121 °C 1×	153.27 ± 6.44 ^d^	12.33 ± 1.89 ^b^	0.25 ± 0.03 ^c^	4.40 ± 0.53 ^c^	1.67 ± 0.31 ^c^
	121 °C 2×	206.55 ± 3.23 ^e^	13.93 ± 2.16 ^b^	0.27± 0.03 ^c^	6.47 ± 0.12 ^d^	2.30 ± 0.44 ^d^
Sah Kecil Island (Kenyir)	24 h	22.80 ± 1.25 ^a^	4.13 ± 1.32 ^a^	0.11 ± 0.01 ^a^	2.23 ± 0.21 ^a^	1.43 ± 0.12 ^a^
	105 °C 1×	67.40 ± 8.97 ^b^	6.30 ± 0.82 ^b^	0.13 ± 0.03 ^b^	3.43 ± 0.35 ^b^	2.57 ± 0.15 ^b^
	105 °C 2×	92.84 ± 27.23 ^b^	7.83 ± 1.12 ^b^	0.16 ± 0.03 ^b^	3.87 ± 0.21 ^b^	2.77 ± 0.15 ^b^
	121 °C 1×	117.38 ± 3.25 ^c^	6.03 ± 1.08 ^b^	0.13 ± 0.01 ^c^	4.50 ± 0.30 ^c^	3.23 ± 0.31 ^c^
	121 °C 2×	146.74 ± 4.00 ^d^	8.97 ± 0.55 ^b^	0.23 ± 0.03 ^d^	5.43 ± 0.47 ^d^	3.47 ± 0.15 ^c^

The values shown as mean with (*n* = 3) followed by ± (standard deviation); NA = not available; ^a,b,c,d,e^ within column, same alphabets indicated no significant difference between treatments within same group using one-way ANOVA (*p* > 0.05) and Tukey post hoc. Comparison between treatments; (24 h—105 °C 1×, 24 h—21 °C 1×, 105 °C 1×–105 °C 2× and 121 °C 1×–121 °C 2×).

**Table 4:** Chemical characterization of dissolved organic matter fractions.

Site	Treatments	Total DOC (mg C/L)	Hydrophobic Fractions (mg C/L)	Hydrophobic Neutrals (mg C/L)	Hydrophilic Fractions (mg C/L)
Chini	24 h	82.54 ± 11.16 ^a^	19.86 ± 7.40 ^a^	5.61 ± 8.09 ^a^	57.08 ± 15.07 ^a^
	105 °C 1×	343.69 ± 23.81 ^b^	80.38 ± 14.28 ^b^	14.34 ± 24.69 ^b^	248.95 ± 4.21 ^b^
	105 °C 2×	444.95 ± 9.90 ^c^	125.19 ± 13.20 ^c^	0.86 ± 1.49 ^c^	325.54 ± 25.07 ^c^
	121 °C 1×	525.83 ± 14.10 ^d^	136.84 ± 10.23 ^d^	29.33 ± 6.34 ^d^	359.66 ± 25.04 ^d^
	121 °C 2×	715.32 ± 31.60 ^e^	200.25 ± 0.76 ^e^	8.48 ± 11.92 ^e^	506.69 ± 29.80 ^e^
Langkawi	24 h	19.53 ± 1.23 ^a^	13.90 ± 1.30 ^a^	1.06 ± 0.93 ^a^	5.36 ± 1.98 ^a^
	105 °C 1×	84.94 ± 6.66 ^b^	33.24 ± 7.93 ^b^	8.81 ± 10.93 ^a^	47.64 ± 20.68 ^b^
	105 °C 2×	101.70 ± 14.1 0 ^b^	45.63 ± 9.13 ^c^	0.16 ± 0.27 ^a^	66.27 ± 7.19 ^b^
	121 °C 1×	151.55 ± 18.51 ^c^	50.91 ± 4.55 ^d^	7.21 ± 12.49 ^a^	104.03 ± 34.93 ^c^
	121 °C 2×	210.93 ± 11.18 ^d^	60.79 ± 2.48 ^d^	0.89 ± 0.99 ^a^	150.13 ± 13.28 ^d^
Kenyir	24 h	42.23 ± 0.90 ^a^	10.43 ± 2.25 ^a^	1.17 ± 1.16 ^a^	31.25 ± 4.40 ^a^
	105 °C 1×	59.81 ± 19.18 ^a^	19.18 ± 3.32 ^a^	8.33 ± 6.03 ^b^	32.30 ± 5.02 ^a^
	105 °C 2×	93.60 ± 43.62 ^a^	26.44 ± 11.33 ^a^	0.00 ± 0.00 ^c^	73.70 ± 31.78 ^b^
	121 °C 1×	105.21 ± 3.80 ^b^	29.07 ± 0.77 ^b^	0.00 ± 0.12 ^d^	79.49 ± 6.96 ^c^
	121 °C 2×	135.18 ± 1.08 ^b^	33.57 ± 1.70 ^b^	0.00 ± 0.00 ^d^	109.20 ± 3.0 *^d^*

The values shown as mean with (*n* = 3) followed by ± (standard deviation). ^a,b,c,d,e^ within row, same low letter case indicated no significant difference between treatments within same group using one-way ANOVA (*p* > 0.05). Comparison between treatments; (24 h–105 °C 1×, 24 h–121 °C 1×, 105 °C 1×–105 °C 2× and 121 °C 1×–121 °C 2×).

**Table 5:** Selected parameters and analytical method.

Parameters	Unit	Analysis Method
pH	-	APHA 4500-H B
Nitrogen, N	%	MS 417L PART 3: 1994
Phosphorus, P	%	MS 678: PART 8
Potassium, K	%	(MS 417: PART 3: 1994): MS 678: PART VI to IX: 1980 (APHA 3500 K))
Total organic carbon	%	MS 678: PART 3A: 1980
Arsenic, As	mg/kg	USEPA 1311 (APHA 3111-B)
Iron, Fe	mg/kg	USEPA 1311 (APHA 3111-B)
Cadmium, Cd	mg/kg	USEPA 1311 (APHA 3111-B)
Chromium, Cr	mg/kg	USEPA 1311 (APHA 3111-B)
Chromium Hexavalent	mg/kg	USEPA 1311 (APHA 3111-B)
Lead, Pb	mg/kg	USEPA 1311 (APHA 3111-B)
Copper, Cu	mg/kg	USEPA 1311 (APHA 3111-B)
Nickel, Ni	mg/kg	USEPA 1311 (APHA 3111-B)
Zinc, Zn	mg/kg	USEPA 1311 (APHA 3111-B)
Mercury, Hg	mg/kg	USEPA 1311 (APHA 3111-B)
Silver, Ag	mg/kg	USEPA 1311 (APHA 3111-B)
**Particle Size Distribution (PSD)**		
Sand	%	USDA/NRCS Soil Taxonomy
Silt	%	USDA/NRCS Soil Taxonomy
Clay	%	USDA/NRCS Soil Taxonomy

**Table 6:** Type of soil extraction methods and the procedure used.

Extraction Methods	Procedure
Natural Extraction 24 Hours (24 h)	Extracted at room temperature for 24 h incubation
Autoclave 105 °C (105 °C)	Extracted at 105 °C using autoclave for 1 h
Autoclave 121 °C (121 °C)	Extracted at 121 °C using autoclave for 1 h
Autoclave 105 °C (105 °C 2×)	Extracted at 105 °C using autoclave for 1 h, cooled (≈30 min) and autoclaved for another 1 h
Autoclave 121 °C (121 °C 2×)	Extracted at 121 °C using autoclave for 1 h, cooled (≈30 min) and autoclaved for another 1 h

## Data Availability

Not applicable.

## Supplementary Materials

The following are available online. Figure S1: Map of sampling sites in this study: (A) Sg. Beruang near Chini Lake, Pahang (3°26′01.3″ N 102°55′08.0″ E); (B) Singa Besar Island, Langkawi Island, Kedah (6°12′51.2″ N 99°44′47.9″ E); and (C) Sah Kecil Island, Kenyir Lake (5°05′01.7″ N 102°48′08.6″ E).

## Sample Availability

Samples of the compounds are not available.

## References

[1] He W., Chen M., Schlautman M.A., Hur J. (2016). Dynamic exchanges between DOM and POM pools in coastal and inland aquatic ecosystems: A review. Sci. Total Environ..

[2] Watanabe M.M., Andersen R.A. (2005). Freshwater Culture media. Algal Culture Techniques.

[3] Marschner B., Kalbitz K. (2003). Controls of bioavailability and biodegradability of dissolved organic matter in soils. Geoderma.

[4] Kalbitz K., Solinger S., Park J.-H., Michalzik B., Matzner E. (2000). Controls on the dynamics of dissolved organic matter in soils: A review. Soil Sci..

[5] Cotrufo M.F., Soong J.L., Horton A.J., Campbell E.E., Haddix M.L., Wall D.H., Parton W.J. (2015). Formation of soil organic matter via biochemical and physical pathways of litter mass loss. Nat. Geosci..

[6] Baldock J., Manning N., Vickerstaff S. (2007). Social Policy.

[7] Kleber M., Sollins P., Sutton R. (2007). A conceptual model of organo-mineral interactions in soils: Self-assembly of organic molecular fragments into zonal structures on mineral surfaces. Biogeochemistry.

[8] Von Lützow M., Kögel-Knabner I., Ludwig B., Matzner E., Flessa H., Ekschmitt K., Guggenberger G., Marschner B., Kalbitz K. (2008). Stabilization mechanisms of organic matter in four temperate soils: Development and application of a conceptual model. J. Plant Nutr. Soil Sci..

[9] Piccolo A. (2002). The supramolecular structure of humic substances: A novel understanding of humus chemistry and implications in soil science. Adv. Agron..

[10] McKee G.A., Soong J.L., Caldéron F., Borch T., Cotrufo M.F. (2016). An integrated spectroscopic and wet chemical approach to investigate grass litter decomposition chemistry. Biogeochemistry.

[11] Huang Y., Eglinton G., Van Der Hage E.R.E., Boon J.J., Bol R., Ineson P. (1998). Dissolved organic matter and its parent organic matter in grass upland soil horizons studied by analytical pyrolysis techniques. Eur. J. Soil Sci..

[12] Hanke D., Dick D.P. (2017). Organic Matter Stocks and the Interactions of Humic Substances with Metals in Araucaria Moist Forest Soil with Humic and Histic Horizons. Rev. Bras. Ciência Solo.

[13] Spaak G. (2014). Investigating the Potential of Dissolved Organic Matter (DOM) Induced Denitrification in Dutch Groundwater. Ph.D. Thesis.

[14] Thurman E.M. (1985). Amount of Organic Carbon in Natural Waters BT-Organic Geochemistry of Natural Waters.

[15] McDonald S., Bishop A.G., Prenzler P.D., Robards K. (2004). Analytical chemistry of freshwater humic substances. Anal. Chim. Acta.

[16] Orlov D. (2020). Humic Substances of Soils and General Theory of Humification.

[17] Essington M.E. (2015). Soil and Water Chemistry: An Integrative Approach.

[18] Schwesig D., Göttlein A., Haumaier L., Blasek R., Ilgen G. (1999). Soil Organic Matter Extraction Using Water at High Temperature and Elevated Pressure (ASE) as Compared to Conventional Methods. Int. J. Environ. Anal. Chem..

[19] Masoom H., Courtier-Murias D., Farooq H., Soong R., Kelleher B.P., Zhang C., Maas W.E., Fey M., Kumar R., Monette M. (2016). Soil Organic Matter in Its Native State: Unravelling the Most Complex Biomaterial on Earth. Environ. Sci. Technol..

[20] Khairil M., Wan Juliana W.A., Nizam M.S., Razi Idris W.M. (2014). Soil properties and variation between three forest types in tropical watershed forest of Chini Lake, Peninsular Malaysia. Sains Malays..

[21] Gasim M.B., Ismail B., Mir S.-I., Rahim S.A., Toriman M.E. (2011). The Physico-chemical Properties of Four Soil Series in Tasik Chini, Pahang, Malaysia. Asian J. Earth Sci..

[22] Rieuwerts J., Thornton I., Farago M., Ashmore M. (1998). Factors influencing metal bioavailability in soils: Preliminary investigations for the development of a critical loads approach for metals. Chem. Speciat. Bioavailab..

[23] Huang B., Li Z., Huang J., Guo L., Nie X., Wang Y., Zhang Y., Zeng G. (2014). Adsorption characteristics of Cu and Zn onto various size fractions of aggregates from red paddy soil. J. Hazard. Mater..

[24] Bech J., Abreu M.M., Chon H.-T., Roca N., Metzler J.B. (2014). Remediation of Potentially Toxic Elements in Contaminated Soils. PHEs, Environment and Human Health.

[25] Kabata-Pendias A. (2010). Trace Elements in Soils and Plants.

[26] Förstner U. (1995). Land Contamination by Metals: Global Scope and Magnitude of Problem in Metal Speciation and Contamination of Soil.

[27] Cui H., Fan Y., Fang G., Zhang H., Su B., Zhou J. (2016). Leachability, availability and bioaccessibility of Cu and Cd in a contaminated soil treated with apatite, lime and charcoal: A five-year field experiment. Ecotoxicol. Environ. Saf..

[28] Asadu C.L.A., Chibuike G.U. (2015). Contributions of organic matter, clay and silt to the effective CEC of soils of different land use history. Adv. Nat. Appl. Sci..

[29] Nciizah A.D., Wakindiki I.I. (2014). Physical indicators of soil erosion, aggregate stability and erodibility. Arch. Agron. Soil Sci..

[30] Moritsuka N., Matsuoka K., Katsura K., Sano S., Yanai J. (2014). Soil color analysis for statistically estimating total carbon, total nitrogen and active iron contents in Japanese agricultural soils. Soil Sci. Plant Nutr..

[31] Molino A., Mehariya S., Di Sanzo G., Larocca V., Martino M., Leone G.P., Marino T., Chianese S., Balducchi R., Musmarra D. (2020). Recent developments in supercritical fluid extraction of bioactive compounds from microalgae: Role of key parameters, technological achievements and challenges. J. CO2 Util..

[32] Da Silva R.P., Rocha-Santos T.A., Duarte A.C. (2016). Supercritical fluid extraction of bioactive compounds. TrAC Trends Anal. Chem..

[33] Berns A.E., Philipp H., Narres H.-D., Burauel P., Vereecken H., Tappe W. (2008). Effect of gamma-sterilization and autoclaving on soil organic matter structure as studied by solid state NMR, UV and fluorescence spectroscopy. Eur. J. Soil Sci..

[34] Teves-Costa P., Oliveira C.S., Senos M.L. (2007). Effects of local site and building parameters on damage distribution in Angra do Heroísmo—Azores. Soil Dyn. Earthq. Eng..

[35] Yaacob N., Ahmad M., Kawasaki N., Maniyam M., Abdullah H., Hashim E., Sjahrir F., Zamri W.W.M., Komatsu K., Kuwahara V. (2021). Kinetics Growth and Recovery of Valuable Nutrients from SE-Langor Peat Swamp and Pristine Forest Soils Using Different Extraction Methods as Potential Microalgae Growth Enhancers. Molecules.

[36] De Oliveira R.L., da Silva O.S., Converti A., Porto T.S. (2018). Thermodynamic and kinetic studies on pectinase extracted from Aspergillus aculeatus: Free and immobilized enzyme entrapped in alginate beads. Int. J. Biol. Macromol..

[37] Chantigny M.H., Curtin D., Beare M.H., Greenfield L.G. (2010). Influence of Temperature on Water-Extractable Organic Matter and Ammonium Production in Mineral Soils. Soil Sci. Soc. Am. J..

[38] Jamien F.M., Embong Z., Tajudin S.A.A., Ahmad S., Lazim A.M. (2018). The optimization of heating temperature for carbon extraction from peat soil. IOP Conf. Ser. Mater. Sci. Eng..

[39] Liegel L.H. (1986). Effects of sterilization procedures on the biological, chemical, and physical properties of soils—A review. Turrialba.

[40] McNamara N., Black H., Beresford N., Parekh N. (2003). Effects of acute gamma irradiation on chemical, physical and biological properties of soils. Appl. Soil Ecol..

[41] Truhlar D.G. (1978). Interpretation of the activation energy. J. Chem. Educ..

[42] Steinweg J.M., Jagadamma S., Frerichs J., Mayes M.A. (2013). Activation Energy of Extracellular Enzymes in Soils from Different Biomes. PLoS ONE.

[43] Inselsbacher E. (2014). Recovery of individual soil nitrogen forms after sieving and extraction. Soil Biol. Biochem..

[44] Martin M., Portetelle D., Michel G., Vandenbol M. (2014). Microorganisms living on macroalgae: Diversity, interactions, and biotechnological applications. Appl. Microbiol. Biotechnol..

[45] Razavi D.S., Lakzian A. (2007). Evaluation of chemical and biological consequences of soil sterilization methods. Casp. J. Environ. Sci..

[46] Wolf B.M., Niedzwiedzki D.M., Magdaong N.C.M., Roth R., Goodenough U., Blankenship R.E. (2017). Characterization of a newly isolated freshwater Eustigmatophyte alga capable of utilizing far-red light as its sole light source. Photosynth. Res..

[47] Dietrich P., Cesarz S., Eisenhauer N., Roscher C. (2000). Effects of steam sterilization on soil abiotic and biotic properties. Soil Org..

[48] Mulani K., Deshpande H. (2018). Humic acid analysis for comparison of soil samples: A forensic perspective. Nucl. Magn. Reson..

[49] DouA S. The thermodynamics stability of soil humic and fulvic acids. Proceedings of the 19th World Congress of Soil Science: Soil Solutions for a Changing World.

[50] Kolokassidou C., Pashalidis I., Costa C., Efstathiou A., Buckau G. (2007). Thermal stability of solid and aqueous solutions of humic acid. Thermochim. Acta.

[51] Fuentes M., González-Gaitano G., García-Mina J.M. (2006). The usefulness of UV–visible and fluorescence spectroscopies to study the chemical nature of humic substances from soils and composts. Org. Geochem..

[52] Francioso O., Montecchio D., Gioacchini P., Ciavatta C. (2005). Thermal analysis (TG–DTA) and isotopic characterization (13C–15N) of humic acids from different origins. Appl. Geochem..

[53] Katsumi N., Yonebayashi K., Okazaki M. (2016). Effects of heating on composition, degree of darkness, and stacking nanostructure of soil humic acids. Sci. Total Environ..

[54] González-Pérez J.A., González-Vila F.J., Almendros G., Knicker H. (2004). The effect of fire on soil organic matter—A review. Environ. Int..

[55] Martín G.A., Vila F.J.G. (2020). Wildfires, soil carbon balance and resilient organic matter in Mediterranean ecosystems. A review. Span. J. Soil Sci..

[56] Page-Dumroese D.S., Harvey A.E., Jurgensen M.F. (1995). A Guide to Soil Sampling and Analysis on the National Forests of the Inland Northwest United States.

[57] Provasoli L., McLaughlin J.J.A., Droop M.R. (1957). The development of artificial media for marine algae. Arch. Microbiol..

[58] Anderson B.H., Magdoff F.R. (2005). Autoclaving Soil Samples Affects Algal-Available Phosphorus. J. Environ. Qual..

[59] Komatsu K., Imai A., Kawasaki N. (2019). Comparison between humic-like peaks in excitation-emission matrix spectra and resin-fractionated humic substances in aquatic environments. Limnology.

[60] Imai A., Fukushima T., Matsushige K., Kim Y.-H., Choi K. (2002). Characterization of dissolved organic matter in effluents from wastewater treatment plants. Water Res..

[61] Leenheer J.A. (1981). Comprehensive approach to preparative isolation and fractionation of dissolved organic carbon from natural waters and wastewaters. Environ. Sci. Technol..

[62] Thurman E.M., Malcolm R.L. (1981). Preparative isolation of aquatic humic substances. Environ. Sci. Technol..

