# Supplementary material for: Assessment of Aqueous Extraction Methods on Extractable Organic Matter and Hydrophobic/Hydrophilic Fractions of Virgin Forest Soils
Source: Molecules. 2021 Apr 23;26(9):2480. doi: 10.3390/molecules26092480 (PMC8123026; doi:10.3390/molecules26092480)
Supplement: Supplementary file 1 [file molecules-26-02480-s001.zip › molecules-1147491-supplementary.pdf]

Assessment of Aqueous Extraction Methods on Extractable Organic Matter and Hydrophobic/Hydrophilic Fractions of Virgin Forest Soils

Wan Muhammad Ikram Wan Mohd Zamri [1], Fridelina Sjahrir [1], Nor Suhaila Yaacob [2,3,*], Noor Fazreen Dzulkafli [1], Mohd Fadzli Ahmad [1], Hasdianty Abdullah [1,3], Maegala Nallapan Maniyam [2,3], Emi Fazlina Hashim [1], Nobuyuki Kawasaki [4], Kazuhiro Komatsu [5] and Victor S. Kuwahara [6]

1 Department of Science & Biotechnology, Faculty of Engineering & Life Sciences, Universiti Selangor, 45600 Bestari Jaya, Selangor, Malaysia; ikramzamri1995@gmail.com (W.M.I.W.M.Z.); fridelina@unisel.edu.my (F.S.); fazreen@unisel.edu.my (N.F.D.); fadzli@unisel.edu.my (M.F.A.); dianty@unisel.edu.my (H.A.); hashim.emifazlina@nies.go.jp (E.F.H.)
2 Institute of Bio-IT Selangor, Universiti Selangor, Jalan Zirkon A7/A, Seksyen 7, 40000 Shah Alam, Selangor, Malaysia; maegala@unisel.edu.my
3 Centre for Foundation and General Studies, Universiti Selangor, Jalan Zirkon A7/A, Seksyen 7, 40000 Shah Alam, Selangor, Malaysia
4 Dainippon Ink and Chemicals DIC Corporation, Central Research Laboratories, 631 Sakado, Chiba 285-8668, Sakura, Japan; nobuyuki-kawasaki@ma.dic.co.jp
5 National Institute for Environmental Studies, 16-2 Onogawa, Ibaraki 305-8506, Tsukuba, Japan;
kkomatsu@nies.go.jp
6 Faculty of Education & Graduate School of Engineering, Soka University, 1-236 Tangi-Machi, Hachioji-Shi 192-8577 Tokyo, Japan; victor@soka.ac.jp
* Correspondence: shuhaila@unisel.edu.my; Tel.: +60-355-223-428

Supplementary Figure S1

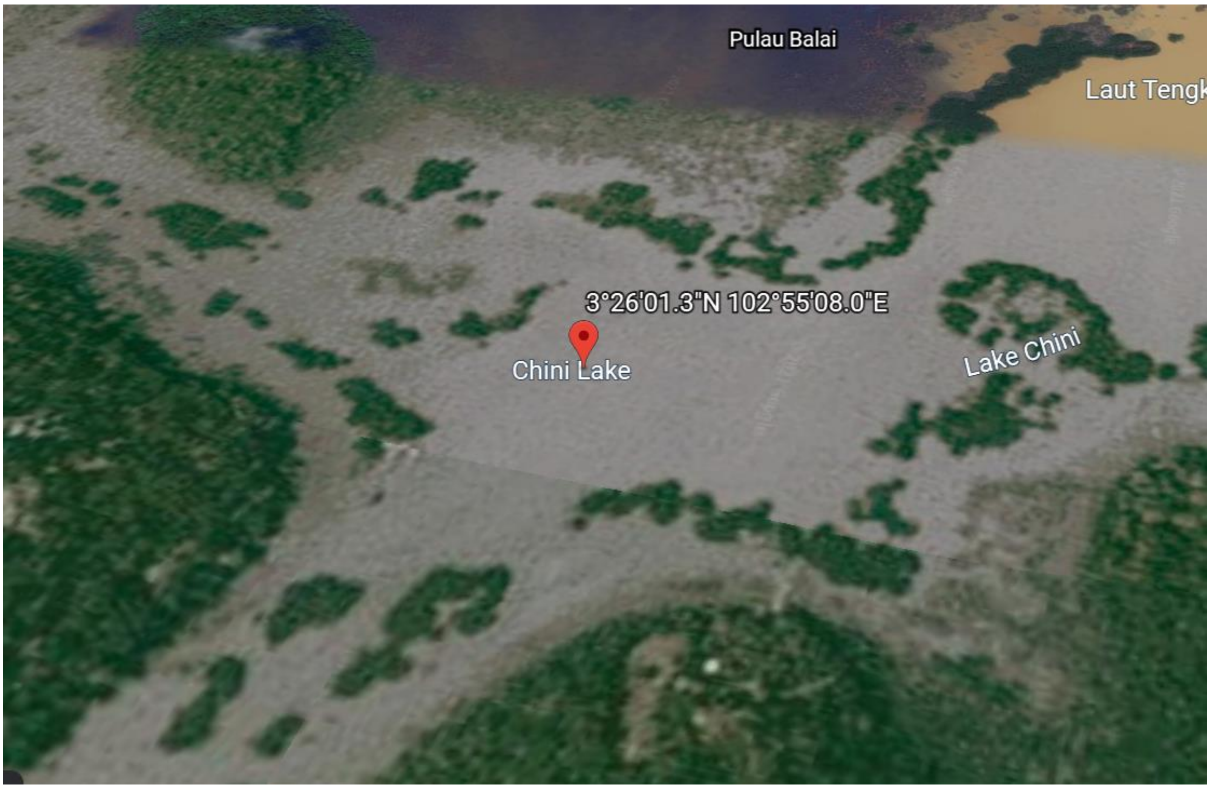

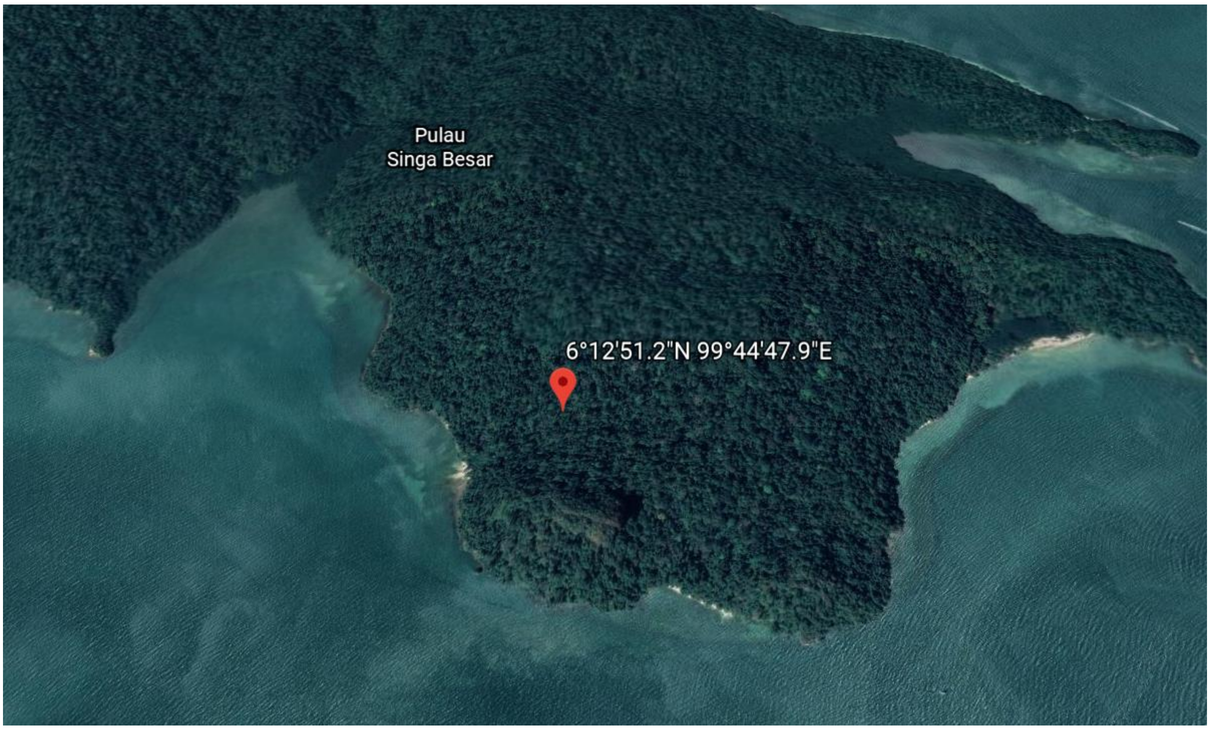

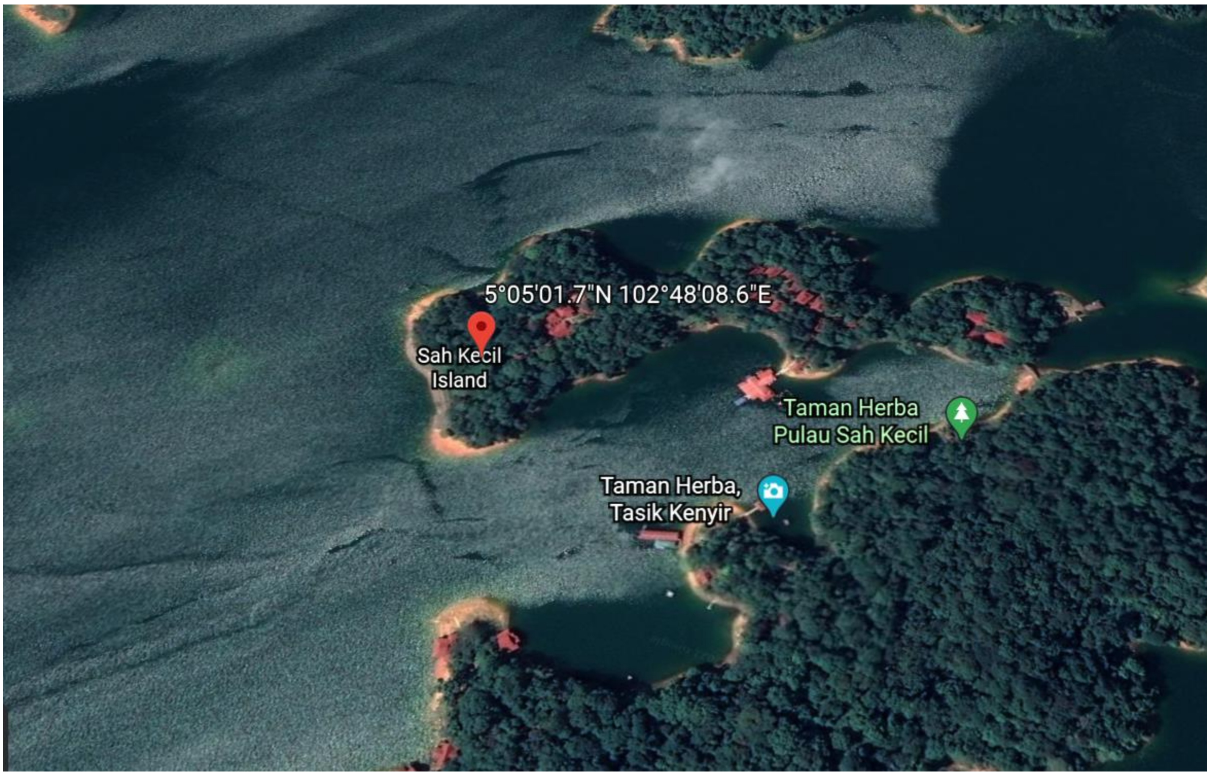

Figure S1: Map of sampling sites in this study A) Sg. Beruang near Chini Lake, Pahang (3°26′′01.3′′N 102°55′08.0′′E) B) Singa Besar Island, Langkawi Island, Kedah (6°12'51.2"N 99°44'47.9"E) and C) Sah Kecil Island, Kenyir Lake ((5°05'01.7"N 102°48'08.6"E).
